# Do cooking and food preparation skills affect healthy eating in college students?

**DOI:** 10.1002/fsn3.3591

**Published:** 2023-08-07

**Authors:** Özge Mengi Çelik, Gizem Aytekin Şahin, Satı Gürel

**Affiliations:** ^1^ Department of Nutrition and Dietetics, Gulhane Faculty of Health Sciences University of Health Sciences Ankara Turkey; ^2^ Department of Nutrition and Dietetics, Faculty of Health Sciences Nuh Naci Yazgan University Kayseri Turkey; ^3^ Department of Nutrition and Dietetics, Faculty of Health Sciences Trakya University Edirne Turkey

**Keywords:** cooking skills, fast‐food, food preparation skills, healthy eating attitude, packaged foods

## Abstract

The aim of this study is to investigate the relationship between food and cooking skills and healthy eating attitudes in college students. The demographic characteristics, anthropometric measurements, nutritional habits, attitudes toward healthy eating, and cooking and food preparation skills were questioned. 16.2% of the students have moderate, 63.5% high, and 20.3% ideally high attitudes toward healthy eating. While a positive and significant correlation was found between the total score and sub‐factor scores of the “Cooking Skills and Food Skills” scale and the total score of the “Attitude Scale for Healthy Nutrition”; a negative statistically significant correlation was found between the total score and sub‐factor scores of the “Cooking Skills and Food Skills” scale and the frequency of consumption of fast‐food, processed meat products, packaged foods, and bread types (*p* < .05). Cooking and food preparation skills seem to be positively associated with healthy attitudes and habits. Considering this positive association, interventions to improve food and cooking skills may help promote healthy eating attitudes in college students. Developing these skills can shed light on increasing the frequency of cooking at home, consuming healthier foods, and as a result, providing a healthy eating attitude. Therefore, more comprehensive intervention studies are needed in this area.

## Graphical abstract

This study aimed to investigate the relationship between food and cooking skills and healthy eating attitudes in college students. A positive correlation was found between the total score and sub‐factor scores of the ‘Cooking Skills and Food Skills’ scale and the total score of the ‘Attitude Scale for Healthy Nutrition’. Cooking and food skills seem to be positively associated with healthy attitudes. Considering this association, interventions to improve food and cooking skills may help promote healthy eating attitudes in students.

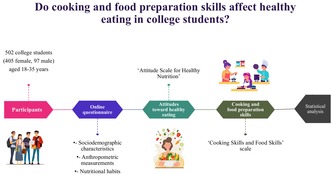

## 1 INTRODUCTION

Healthy nutrition is essential at every stage of life to protect and develop health and increase the quality of life. It is stated that inadequate and unbalanced nutrition causes obesity and other non‐communicable diseases related to nutrition (such as Type II diabetes, cardiovascular diseases, and some types of cancer; Besler et al., 2015). A healthy and balanced diet requires a wide range of skills in planning meals and purchasing and preparing food (Vidgen & Gallegos, 2014). Studies report that consumption of home‐prepared foods is associated with better diet quality and weight control (Clifford Astbury et al., 2019b; Van der Horst et al., 2011; Wolfson et al., 2020; Wolfson & Bleich, 2015). While meals consumed outside are high in saturated fat, cholesterol, salt, and energy, vitamin, mineral, and fiber content are low. Therefore, increased frequency of eating out was associated with increased body weight and decreased diet quality (Herbert et al., 2014; Laska et al., 2012). In addition, other studies have found that the fast‐food consumption of individuals who do not like to cook increases (Dave et al., 2009) and that there is a negative relationship between the skill and behavior of preparing meals at home and the consumption of excessively processed foods (Lam & Adams, 2017). Therefore, interventions to improve individuals' cooking and food preparation skills have become popular in public health (McGowan et al., 2017).

*Cooking skills* are defined as “the set of mechanical or physical skills used in food preparation.” This definition includes the conceptual and perceptual skills associated with changes during food cooking. *Food preparation skills* include reading labels, meal planning, purchasing ingredients, preparing, cooking, and budgeting (Lavelle et al., 2017; McGowan et al., 2016, 2017). Studies show that a sustainable healthy diet can be associated with the ability to prepare and cook food (Clifford Astbury et al., 2019a; Condrasky et al., 2011). However, it is reported that there is a decrease in cooking skills and food preparation rates at home due to factors such as workload, daily stress, and ease of access to ready‐to‐eat foods, especially in Western societies (Herbert et al., 2014). Acquiring and developing skills in the kitchen, greater food preparation self‐efficacy, increased interest in cooking activities, and increased frequency of home cooking are important for healthy eating. Therefore, food preparation and cooking skills interventions have proliferated, and some countries have mentioned home cooking in their dietary guidelines (Clifford Astbury et al., 2019a; Condrasky et al., 2011).

Diet quality is influenced by many factors, such as nutritional knowledge, beliefs, and attitudes, in addition to food preparation and cooking skills (Aggarwal et al., 2014; Freeland‐Graves & Nitzke, 2013). Attitudes toward nutrition and healthy eating affect food choices and, thus, diet quality (Aggarwal et al., 2014; Lê et al., 2013).

Transitioning to young adulthood is an important period for acquiring and strengthening cooking and meal preparation skills and developing attitudes toward healthy eating (Laska et al., 2012). Furthermore, acquiring these skills and attitudes early is also crucial for sustainable healthy eating habits (Lavelle et al., 2016). However, it has been determined that many college students with independent parental control have low awareness of healthy eating (Pawlak et al., 2009). In addition, studies have reported that attitude toward healthy eating is one factor affecting college students' food choices (Aggarwal et al., 2014). However, as far as we know, there has yet to be a study investigating the effects of cooking and food preparation skills on healthy eating attitudes among university students in Turkey. For this reason, this study aimed for the first time to evaluate the cooking and food preparation skills and attitudes toward healthy eating in Turkish college students and to associate them with some factors.

## 2 MATERIALS AND METHODS

This descriptive and cross‐sectional study was conducted with 502 college students (405 female, 97 male) aged 18–35 studying at Trakya University Faculty of Health Sciences between February and June 2022. All volunteer students who agreed to participate in the study were included in the study. Before starting the study, ethical approval with the decision number 01/11 dated January 3, 2022, from Trakya University Faculty of Medicine Dean's Office of Ethics Committee for Non‐Invasive Scientific Research. All procedures in the study were carried out in accordance with the Declaration of Helsinki. Research data were collected using a web‐based questionnaire. Researchers created data collection tool through Google surveys. The online questionnaire was sent to the students' e‐mails. The sample of the study was formed by the individuals who ticked the “I consent to participate in this study voluntarily” tab at the beginning of the form and filled out the questionnaire completely.

The questionnaire consisted of five parts: (1) sociodemographic characteristics (gender, age, grade of education, and place of residence), (2) anthropometric measurements (body weight and height), (3) nutritional habits, (4) attitudes toward healthy eating, and (5) cooking and food preparation skills.

### 2.1 Attitudes toward healthy eating

Individuals' attitudes toward healthy eating were evaluated using the “Attitude Scale for Healthy Nutrition.” The scale was developed and validated in Turkish by Tekkurşun Demir and Cicioğlu (Demir & Cicioğlu, 2019) in 2019. The Cronbach's alpha internal consistency coefficient of the scale is 0.90. The scale consists of 21 questions and has four sub‐factors information on nutrition, emotion for nutrition, positive nutrition, and malnutrition. The scale is five‐point Likert type. The ratings for the positive items on the scale are “Strongly Disagree,” “Disagree,” “Undecided,” “Agree,” and “Strongly Agree.” Positive attitude items are scored as 1, 2, 3, 4, and 5, while negative attitude items are scored as 5, 4, 3, 2, and 1. The lowest score that can be obtained from the scale is 21, and the highest score is 105. An increase in the score obtained from the scale indicates that individuals have a higher level of attitudes toward healthy eating. In the original scale, quarters were used to find the cut‐off point, and the scale scores were defined as “low, moderate, high, ideally high” using cut‐off points. Weighted Kappa statistics were used for the reliability of the multi‐category scale. 21–42 points indicate a low healthy eating attitude, 43–63 points moderate healthy eating attitude, 64–84 points high healthy eating attitude, and 85–105 points ideally high healthy eating attitude.

### 2.2 Cooking and food preparation skills

Individuals cooking and food preparation skills were evaluated using the “Cooking Skills and Food Skills” scale. This scale was developed by Lavelle et al. (2017). The Turkish reliability and validity of the scale were done by Keleş & Akçil (2021). The Cronbach's alpha internal consistency coefficient of the scale is 0.954. The scale consists of 33 questions and has two sub‐factors: cooking skills and food skills. The cooking skills sub‐factor consists of 14 items and the food skills sub‐factor consists of 19 items. The scale is scored with an 8‐point Likert system ranging from 1 (very bad) to 7 (very good) with never/rarely option. Participants are asked to rate how good they are at each skill, and scores between 1 and 7 are added for the skills used. Participants who tick never/rarely get 0 points. The cooking skills score ranges from 0 to 98 points, the food skills score ranges from 0 to 133 points, and Cooking Skills and Food Skills total score ranges from 0 to 231 points. An increase in the total score obtained from the scale indicates that cooking and food preparation skills are high.

### 2.3 Nutritional habits

In the online questionnaire, consumption of main meals and snacks, the frequency of meals outside, and the consumption frequency of fast‐food, processed meat products, packaged foods, and bread types were questioned. Consumption 3 times a week or more was classified as “very often,” consumption 1–2 times a week as “frequent,” consumption once in 15 days and once a month as “rarely,” and no consumption as “never.”

### 2.4 Anthropometric measurements

Height and body weight measurements were taken based on the self‐reports of individuals. Individuals were informed about how to take anthropometric measurements in the questionnaire form. The body mass index (BMI) value was calculated by dividing the body weight by the square of the height. Body mass index below 18.50 kg/m^2^ was classified as underweight, between 18.50 and 24.99 kg/m^2^ as normal, between 25.0 and 29.99 kg/m^2^ as overweight, and above 30.0 kg/m^2^ as obese (Gibson, 2005).

### 2.5 Statistical analysis

The G*Power (version 3.1.9.7, Universitat Düsseldorf, Düsseldorf, Germany) was used for post‐hoc power analysis, and the effect size was calculated for the correlation between the total score of the “Attitude Scale for Healthy Nutrition” and the total score of the “Cooking Skills and Food Skills” scale. According to the analysis, the study power (1−β) was 99% for the statistical significance of two‐sided alpha of 5%.

The Statistical Package for the Social Sciences (version 22.0) software was used for all analyses. The normal distribution of the data was examined using visual (histogram and probability graphs) and analytical methods (Shapiro–Wilk). Descriptive statistics were given as frequency and percentage values for categorical variables and mean and standard deviation for numerical variables. These tests showed that all numerical variables did not fit into a normal distribution. Therefore, non‐parametric statistical tests were used. Kruskal Wallis and Mann–Whitney U tests were used in independent groups for comparison. Bonferroni correction was applied for multiple pairwise comparisons. Regression analysis was performed for the relationship between attitudes toward healthy eating and cooking and food skills. For the simple linear regression analysis, the non‐normally distributed variables were transformed using logarithmic transformation to more closely approximate normal distribution. Relationships between numerical variables were given with the Spearman correlation coefficient. A *p*‐value of less than .05 was considered to be statistically significant.

## 3 RESULTS

The mean age of the individuals was 20.8 ± 2.32 years. 18.9% of the students were in the first grade, 32.7% in the second, 20.7% in the third, 23.3% in the fourth, and 4.4% in the fifth. More than half of the students (57.6%) lived in the dormitory. The mean BMI of the students was 21.8 ± 3.55 kg/m^2^. The majority of individuals (72.3%) were within the normal range according to BMI classification. The students' mean number of main meals was 2.5 ± 0.52, and the number of snacks was 1.5 ± 0.89. While 32.5% of the students stated that they did not consume their breakfast meal outside, most of them consumed lunch (62.7%) and dinner meals (47.2%) often outside. 16.2% had moderate, 63.5% high, and 20.3% ideally high attitudes toward healthy eating. More than half of the individuals stated that they consume fast‐food, processed meat products, packaged foods, and types of bread often and very often (Table 1).

**TABLE 1:** General characteristics of individuals.

Variables	*N* (%)
Gender	
Female	405 (80.7%)
Male	97 (19.3%)
Grade of education	
1st	95 (18.9%)
2nd	164 (32.7%)
3rd	104 (20.7%)
4th	117 (23.3%)
5th	22 (4.4%)
Place of residence	
With family at home	113 (22.5%)
Alone at home	48 (9.6%)
With friends at home	52 (10.4%)
In the dormitory	289 (57.6%)
BMI classification	
Underweight (<18.50 kg/m^2^)	71 (14.1%)
Normal (18.50–24.99 kg/m^2^)	363 (72.3%)
Overweight (25.00–29.99 kg/m^2^)	49 (9.8%)
Obese (≥30.0 kg/m^2^)	19 (3.8%)
Frequency of eating meals out	
Breakfast	
Very often	55 (11.0%)
Often	66 (13.1%)
Rarely	218 (43.4%)
Never	163 (32.5%)
Lunch	
Very often	193 (38.4%)
Frequent	122 (24.3%)
Rarely	121 (24.1%)
Never	66 (13.1%)
Dinner	
Very often	93 (18.5%)
Frequent	144 (28.7%)
Rarely	201 (40.0%)
Never	64 (12.7%)
Frequency of fast‐food consumption	
Very often	110 (21.9%)
Frequent	151 (30.1%)
Rarely	223 (44.4%)
Never	18 (3.6%)
Frequency of processed meat products consumption	
Very often	141 (28.1%)
Frequent	124 (24.7%)
Rarely	178 (35.5%)
Never	59 (11.8%)
Frequency of packaged foods consumption	
Very often	199 (39.6%)
Often	141 (28.1%)
Rarely	138 (27.5%)
Never	24 (4.8%)
Frequency of types of bread consumption	
Very often	131 (26.1%)
Often	136 (27.1%)
Rarely	209 (41.6%)
Never	26 (5.2%)
Evaluation of attitude scale for healthy nutrition	
Moderate	81 (16.2%)
High	319 (63.5%)
Ideally high	102 (20.3%)
	$\bar{X}$ ± SD
Age (years)	20.8 ± 2.32
BMI (kg/m^2^)	21.8 ± 3.55
Number of main meals	2.5 ± 0.52
Number of snacks	1.5 ± 0.89
Attitude Scale for Healthy Nutrition total score	75.1 ± 11.10
Sub‐factors	
Information on nutrition	21.0 ± 4.29
Emotion for nutrition	17.1 ± 4.74
Positive nutrition	17.7 ± 4.51
Malnutrition	19.1 ± 4.56

Abbreviation: BMI, body mass index.

A statistically significant difference was found between the genders regarding the total score of the “Attitude Scale for Healthy Nutrition” (*p* < .05). A statistically significant difference was found between the grades of education in terms of the total score of the “Attitude Scale for Healthy Nutrition,” sub‐factor scores of information on nutrition, and positive nutrition (*p* < .05). A statistically significant difference was found in the positive nutrition sub‐factor score according to the BMI classification (*p* < .05; Table 2). The total score of the “Attitude Scale for Healthy Nutrition” in the first grade is lower than that in the third and fourth grades (*p* < .005). The score of information on nutrition in the first grade is lower than in all other grades (*p* < .005). The positive nutrition score in the first grade is lower than in the fourth grade (*p* < .005). The positive nutrition score of obese individuals is lower than other individuals (*p* < .0083; statistics obtained as a result of Bonferroni correction are not shown in the table.)

**TABLE 2:** Evaluation of individuals' attitudes toward healthy eating according to some variables.

	Sub‐factors									
	Attitude scale for healthy nutrition total score ($\bar{X}$ ± SS)	*p*‐value	Information on nutrition score ($\bar{X}$ ± SS)	*p*‐value	Emotion for nutrition score ($\bar{X}$ ± SS)	*p*‐value	Positive nutrition score ($\bar{X}$ ± SS)	*p*‐value	Malnutrition score ($\bar{X}$ ± SS)	*p*‐value
Gender										
Female	75.5 ± 11.06	**.023** ^a^ ^,^ *	21.2 ± 4.00	.207^a^	17.2 ± 4.51	.129^a^	17.8 ± 4.33	.599^a^	19.2 ± 4.50	.366^a^
Male	73.1 ± 11.13		20.2 ± 5.31		16.8 ± 5.62		17.3 ± 5.21		18.7 ± 4.79	
Grade of education										
1st	71.7 ± 8.68^x^	**.002** *	18.6 ± 5.19^x^	**<.001** *	17.7 ± 5.46	.0.88	16.1 ± 4.88^x^	**.003** *	19.2 ± 4.46	.875
2nd	74.8 ± 11.75		20.6 ± 4.27^y^		17.2 ± 4.49		17.8 ± 4.40		19.0 ± 4.88	
3rd	76.6 ± 10.05^y^		22.0 ± 3.89^z^		17.3 ± 4.29		17.9 ± 3.97		19.3 ± 4.38	
4th	77.0 ± 12.55^y^		22.5 ± 3.00^z^		16.9 ± 4.92		18.5 ± 4.74^y^		18.8 ± 4.59	
5th	73.2 ± 8.80		21.7 ± 2.77^z^		14.7 ± 3.82		17.7 ± 3.46		18.9 ± 3.25	
Place of residence										
With family at home	76.7 ± 11.81	.101	21.0 ± 4.41	.430	17.7 ± 4.83	.555	18.1 ± 4.90	.252	19.9 ± 4.14	.127
Alone at home	76.6 ± 12.56		22.1 ± 3.07		17.3 ± 5.49		18.3 ± 4.45		18.8 ± 4.49	
With friends at home	72.6 ± 10.35		20.8 ± 4.31		16.7 ± 3.93		17.0 ± 4.50		18.0 ± 5.19	
In the dormitory	74.5 ± 10.61		20.9 ± 4.41		17.0 ± 4.71		17.6 ± 4.36		19.0 ± 4.58	
BMI classification										
Underweight	76.2 ± 10.92	.318	22.0 ± 3.49	.180	17.0 ± 4.67	.981	18.2 ± 4.11^x^	**.013** *	18.9 ± 4.78	.171
Normal	75.0 ± 11.03		20.8 ± 4.39		17.2 ± 4.65		17.6 ± 4.51^x^		19.3 ± 4.41	
Overweight	74.9 ± 12.27		21.2 ± 4.29		16.9 ± 5.43		18.5 ± 5.09^x^		18.1 ± 5.38	
Obese	70.7 ± 9.72		20.6 ± 4.93		17.0 ± 5.17		15.2 ± 3.56^y^		17.8 ± 3.97	

Abbreviation: BMI, body mass index.

^a^ Mann–Whitney U test, other tests Kruskal Wallis test, there is a significant difference between x, y, z groups.

* *p* < .05 (Bold).

A statistically significant difference was found between the genders in terms of the total score and sub‐factor scores of the “Cooking Skills and Food Skills” scale (*p* < .05). In addition, statistically significant difference was found between the grades of education in terms of the total score of “Cooking Skills and Food Skills” scale and the sub‐factor score of the cooking skills (*p* < .05; Table 3). The total score of the “Cooking Skills and Food Skills” scale and the cooking skills score in the first grade were lower than in the second grade (*p* < .005; statistics obtained as a result of Bonferroni correction are not shown in the table.)

**TABLE 3:** Evaluation of individuals' cooking and food preparation skills.

	Sub‐factors					
	Cooking skills and food skills total score	*p*‐value	Cooking skills score	*p*‐value	Food skills score	*p*‐value
Gender						
Female	155.8 ± 39.62	<**.001** ^a^ ^,^ *	65.7 ± 19.38	<**.001** ^a^ ^,^ *	90.1 ± 24.67	**.004** * ^,^ ^a^
Male	136.3 ± 50.86		55.7 ± 24.50		80.6 ± 30.24	
Grade of education						
1st	137.6 ± 49.76^x^	**.042** *	57.2 ± 22.93^x^	**.023** *	80.4 ± 30.19	.078
2nd	156.2 ± 42.54^y^		65.7 ± 21.55^y^		90.5 ± 26.69	
3rd	154.3 ± 39.20^y^		65.9 ± 18.94^y^		88.4 ± 24.01	
4th	154.5 ± 37.74^y^		63.5 ± 18.98^y^		90.9 ± 22.48	
5th	158.7 ± 41.36^y^		68.9 ± 19.01^y^		89.7 ± 25.02	
Place of residence						
With family at home	150.1 ± 47.37	.537	62.8 ± 23.59	.464	87.2 ± 27.91	.791
Alone at home	160.2 ± 40.15		68.8 ± 19.72		91.43 ± 24.77	
With friends at home	151.4 ± 36.41		64.0 ± 18.18		87.40 ± 22.93	
In the dormitory	151.5 ± 42.23		63.3 ± 20.27		88.2 ± 26.16	
BMI classification						
Underweight	154.4 ± 38.75	.937	64.2 ± 19.97	.979	90.1 ± 23.24	.831
Normal	151.8 ± 41.95		63.9 ± 20.25		87.8 ± 25.79	
Overweight	151.4 ± 45.50		62.4 ± 23.69		88.9 ± 26.48	
Obese	148.8 ± 62.30		62.4 ± 27.60		85.8 ± 39.32	

Abbreviation: BMI, body mass index.

^a^ Mann–Whitney U test, other tests Kruskal Wallis test, there is a significant difference between x, y groups.

* *p* < .05 (Bold).

There was a positive correlation between the total score of the “Cooking Skills and Food Skills” scale and the grade of the education class, the total score of the “Attitude Scale for Healthy Nutrition,” sub‐factor scores of information on nutrition, positive nutrition, and malnutrition; there was a negative correlation between the total score of the “Cooking Skills and Food Skills: scale and the frequency of consumption of fast‐food, processed meat products, packaged foods, and bread types; there was a positive correlation between cooking skills score and the total score of the “Attitude Scale for Healthy Nutrition,” sub‐factor scores of information on nutrition and positive nutrition; there was a negative correlation between cooking skills score and the frequency of consumption of fast‐food, processed meat products, packaged foods, and bread types; there was a positive correlation between food skills score and the total score of the “Attitude Scale for Healthy Nutrition,” sub‐factor scores of information on nutrition, positive nutrition, and malnutrition; there was a negative correlation between food skills score and the frequency of consumption of fast‐food, processed meat products, packaged foods, and bread types (*p* < .05; Table 4).

**TABLE 4:** Evaluation of the relationship between cooking and food preparation skills and some variables.

	Cooking skills and food skills total score	Cooking skills score	Food skills score
Age (years)	*r* = .049	*r* = .058	*r* = .035
	*p* = .278	*p* = .193	*p* = .435
BMI (kg/m^2^)	*r* = −.032	*r* = −.002	*r* = −.048
	*p* = .481	*p* = .963	*p* = .285
Grade of education	*r* = .089	*r* = .081	*r* = .083
	*p* = .047*	*p* = .070	*p* = .064
Number of main meals	*r* = .016	*r* = −.010	*r* = .032
	*p* = .720	*p* = .825	*p* = .471
Number of snacks	*r* = .023	*r* = .013	*r* = .020
	*p* = .611	*p* = .770	*p* = .651
Attitude Scale for Healthy Nutrition total score	*r* = .291	*r* = .224	*r* = .316
	*p* < .001*	*p* < .001*	*p* < .001*
Sub‐factors			
Information on nutrition	*r* = .315	*r* = .278	*r* = .313
	*p* < .001*	*p* < .001*	*p* < .001*
Emotion for nutrition	*r* = .056	*r* = .021	*r* = .068
	*p* = .213	*p* = .641	*p* = .128
Positive nutrition	*r* = .276	*r* = .248	*r* = .269
	*p* < .001*	*p* < .001*	*p* < .001*
Malnutrition	*r* = −.118	*r* = −.058	*r* = −.162
	*p* = .008*	*p* = .196	*p* < .001*
Frequency of eating meals out			
Breakfast	*r* = −.014	*r* = .002	*r* = −.023
	*p* = .761	*p* = .968	*p* = .608
Lunch	*r* = −.049	*r* = −.052	*r* = −.027
	*p* = .277	*p* = .246	*p* = .540
Dinner	*r* = −.021	*r* = .011	*r* = −.043
	*p* = .638	*p* = .809	*p* = .335
Frequency of fast‐food consumption	*r* = −.138	*r* = −.105	*r* = −.142
	*p* = .002*	*p* = .019*	*p* = .001*
Frequency of processed meat products consumption	*r* = −.116	*r* = −.088	*r* = −.124
	*p* = .010*	*p* = .048*	*p* = .005*
Frequency of packaged foods consumption	*r* = −.131	*r* = −.097	*r* = −.140
	*p* = .003*	*p* = .029*	*p* = .002*
Frequency of types of bread consumption	*r* = −.145	*r* = −.112	*r* = −.151
	*p* = .001*	*p* = .012*	*p* = .001*

*Note*: Spearman correlation, *p < .05 (Bold).

Abbreviation: BMI, body mass index.

When the factor that could affect the total score of the “Attitude Scale for Healthy Nutrition” was evaluated with simple linear regression analysis, the model was deemed significant (*R* ^2^ = 0.311; *p* < .001). It was determined that total score of the “Cooking Skills and Food Skills” scale had an effect on attitude toward healthy eating. Higher “Cooking Skills and Food Skills” scale total score was associated with a higher “Attitude Scale for Healthy Nutrition” total score (*p* < .05; Table 5).

**TABLE 5:** Simple linear regression model for the relationship between attitude toward healthy eating and cooking and food skills.

Model	Attitude scale for healthy nutrition total score		
	Beta	*t*	*p*
Cooking skills and food skills score	0.311	7.313	<.001*
		***R*** ^**2**^ = 0.311; *p* < .001*	

* *p* < .05 (Bold).

## 4 DISCUSSION

To the best of our knowledge, this is the first study that evaluated the effects of Turkish college students' cooking and food skills on attitudes toward healthy eating. The primary findings of this study have shown that greater cooking and food skills scores were associated with a higher attitude toward healthy eating. Moreover, the present study showed a negative correlation between both “Cooking and Food Skills” total score and sub‐factor scores and the frequency of consumption of fast‐food, processed meat, packaged food, and bread types. Considering the increasing rates of diet‐related chronic diseases due to unhealthy nutrition, we thought that these findings might shed light on the importance of interventions to improve cooking and food skills in the young population.

Unhealthy eating patterns have been associated with increased rates of non‐communicable diseases (such as obesity and diabetes; Cooke & Papadaki, 2014). In addition, adopting healthy dietary practices during transitioning from adolescence to adulthood is especially important for reducing the risk of chronic disease later in life (Steptoe et al., 2002). Studies conducted with university students in different countries have shown that students' consumption of vegetables and fruits is low, on the contrary, their consumption of high‐fat foods, added sugar, and alcohol is high (El Ansari et al., 2012; Moreno‐Gómez et al., 2012; Tanton et al., 2015). Briefly, studies have shown that college students often adopt an unhealthy dietary pattern. Therefore, it is important to evaluate college students' nutritional habits and attitudes toward healthy eating during this period.

A healthy and balanced diet requires a wide range of skills in planning meals, purchasing, and preparing food (Vidgen & Gallegos, 2014). Studies reported that consumption of home‐prepared foods is associated with better diet quality and weight control (Clifford Astbury et al., 2019b; Van der Horst et al., 2011; Wolfson et al., 2020; Wolfson & Bleich, 2015). However, in this study, we approached the subject from a different perspective and focused on the effect of food and cooking skills on healthy eating attitudes in college students.

In the current study, it was determined that higher cooking and food skill total scores in college students were associated with higher attitudes toward healthy eating. In addition, the “Cooking and Food Skills” total score and sub‐factor scores were found to be positively correlated with “information on nutrition” and “positive nutrition” sub‐factor scores, which are sub‐factors of the “Attitude Scale for Healthy Nutrition.” Furthermore, total score of “Cooking Skills and Food Skills” and food skills sub‐factor score was negatively correlated with “malnutrition.” The malnutrition sub‐factor in the “Attitude Scale for Healthy Nutrition” included negative statements such as skipping meals and consuming junk food. “Information on Nutrition” and “Positive Nutrition” sub‐factors include knowledge and positive attitudes about healthy nutrition (Demir & Cicioğlu, 2019). These findings may indicate that participants with high cooking and eating skills are more aware of healthy eating and have a higher healthy eating attitude. In the literature, developing food and cooking skills and cooking at home more frequently has been associated with a healthier diet (Hagmann et al., 2020; Hartmann et al., 2013; Wolfson & Bleich, 2015). In addition, many intervention studies have shown that improving cooking skills positively affects food choices (Reicks et al., 2018). Our findings are in line with these results.

Previous studies have often shown that college students lack the knowledge and skills to make healthy food choices and prepare healthy food (Allom & Mullan, 2014; Cluskey & Grobe, 2009). It has been stated that college students getting away from their parents and gaining self‐control may affect this situation (Deliens et al., 2014). In the literature, the transition from home to college life has been associated with negative changes in food intake, such as an increase in alcohol and sugar intake, a decrease in fruit and vegetable consumption, and an increase in fast‐food and processed food consumption (Papadaki et al., 2007; Tanton et al., 2015). In the current study, more than half of the students stated that they “often” and “very often” consume fast‐food, processed meat products, packaged foods, and different types of bread. It is challenging to comment on the frequency of food consumption for university students as we lack information regarding their food preferences before getting into college. However, many factors, such as changes in living conditions, budgetary constraints, and greater accessibility to fast‐food, may lead college students to consume these foods more (Papadaki et al., 2007). Moreover, a negative correlation was found between the consumption frequency of these products and the “Cooking Skills and Food Skills” scale scores. These results were in line with other study findings showing a negative association between home‐preparation skills and behavior and consumption of fast‐food and highly processed foods (Dave et al., 2009; Lam & Adams, 2017).

Previous studies have reported that females score higher on the “Cooking Skills and Food Skills” scale (Hartmann et al., 2013; McGowan et al., 2016), and there was a stronger correlation between cooking skills and healthy eating, especially in females (Hartmann et al., 2013; McGowan et al., 2017). In our study, as in other studies, the total score on the “Cooking Skills and Food Skills” scale was significantly higher in females than in men. The fact that females are more responsible for cooking and preparing foods at home compared to men may be effective in higher scores. Moreover, in the current study, it was determined that the total score on the “Attitude Scale for Healthy Nutrition” of females was also higher than that of men. According to our findings, the fact that females' cooking and cooking skills and their attitudes toward healthy eating are higher than males, confirming our hypothesis that there is a relationship between these two parameters. Similarly, in previous studies, it was determined that women's interests and attitudes toward healthy nutrition were higher than men's (Roininen et al., 2001; Sümen & Evgin, 2022).

This study showed that the “Cooking Skills and Food Skills” total score and the “Cooking Skills” scores of the fifth‐grade students were higher than the first‐grade students. Our participants were students studying at the faculty of health sciences. Therefore, the increase in nutrition and health‐related courses as the grades increase in the curriculum may have caused this situation. Another reason may be that students adapt to living away from their parents.

Attitudes toward healthy eating influence food choices and, thus, diet quality (Aggarwal et al., 2014; Lê et al., 2013). In the current study, the majority of the students had high (63.5%) and ideally high (20.3%) scores on the “Attitudes Scale for Healthy Eating.” Similarly, in another study conducted on adults in Turkey, 54.6% of the participants were found to have high, and 31% had ideally high eating attitudes (Özenoğlu et al., 2021). Moreover, in our study, it was observed that the “Attitude Scale for Healthy Nutrition” total score, “Information on Nutrition” score, and “Positive Nutrition” score of the students increased with the increase in the grade of education. Based on these findings, it can be said that university students' attitudes toward healthy eating, similar to their food preparation and cooking skills, are worse in the first years when they are away from their families, but they improve later on depending on age and perhaps university education.

Positive nutrition attitudes promote healthy eating habits (Stroebele‐Benschop et al., 2018). In addition, Mötteli & Hotzy (2022) found that cooking and food skills were highly correlated with a positive attitude toward a healthy diet. In our study, the positive nutrition attitudes of obese individuals were significantly lower. Other studies also found that participants with low/normal BMI were more likely to have more positive attitudes toward nutrition than those overweight/obese participants (Acheampong & Haldeman, 2013; Sümen & Evgin, 2022). In addition, positive attitudes toward healthy nutrition were associated with healthier diets, such as lower energy density and more fruit and vegetable servings (Aggarwal et al., 2014). Briefly, since a positive nutrition attitude affects diet quality (Aggarwal et al., 2014; Lê et al., 2013), it may be expected to be lower in obese participants.

The present study has several strengths. This is the first study to reveal the relationship between food preparation and cooking skills and healthy nutrition in Turkish college students. The study emphasizes the importance of food preparation and cooking skills for displaying an attitude toward healthy eating. For individuals to maintain healthy eating habits, their cooking and food preparation skills must also be high. Insufficient food preparation and cooking skills lead to unhealthy food preferences, and thus the frequency of consumption of these foods increases. More attention should be paid to this issue in terms of non‐communicable chronic diseases in college students, who are young adults.

Nevertheless, there are some limitations to our study. First, because of the study's cross‐sectional nature, we cannot provide a causal link between cooking and food skills and healthy eating attitudes. Second, since the current study's findings were based on the participants' self‐report data, there may be a risk of resource bias. Another limitation is the lack of information about the education that parents give to their children in food preparation and cooking, and how much exposure the participants were exposed to cooking at home or during growing up. Finally, participation in the study was not equal between the genders. Nonetheless, we believe that the present study may shed light on future studies as it is the first time it has evaluated Turkish college students' cooking and food skills and their attitudes toward healthy eating. However, it may be beneficial to include not only college students but also the general population in future studies.

## 5 CONCLUSION

To our knowledge, this is the first study to evaluate the effect of cooking and food skills on healthy eating attitudes in Turkish college students. The main findings of our study showed that higher cooking and food skills were associated with higher healthy eating attitudes. In conclusion, cooking and food skills seem to be associated with healthy eating attitudes and eating habits. Considering this positive association, interventions to improve food and cooking skills may help promote healthy eating attitudes in college students. Developing these skills can shed light on increasing the frequency of cooking at home, consuming healthier foods and as a result, providing a healthy eating attitude. Therefore, more comprehensive intervention studies are needed in this area.

## AUTHOR CONTRIBUTIONS

**Özge Mengi Çelik:** Conceptualization (equal); funding acquisition (equal); methodology (equal); project administration (equal); supervision (equal); writing – original draft (equal); writing – review and editing (equal). **Gizem Aytekin Şahin:** Conceptualization (equal); supervision (equal); writing – review and editing (equal). **Satı Gürel:** Conceptualization (equal); supervision (equal); writing – review and editing (equal).

## Data Availability

The datasets generated and analyzed during the current study are available from the corresponding author on a reasonable request.
